# Effects of Extracorporeal Shockwave Therapy on Wound Healing in Rats

**DOI:** 10.7759/cureus.83369

**Published:** 2025-05-02

**Authors:** Takuro Sugiyama, Hidetaka Murakami, Shinichiro Tanaka, Shiro Yoshida, Takahiro Sato, Koji Hiraoka

**Affiliations:** 1 Department of Orthopedic Surgery, Kurume University, Fukuoka, JPN; 2 Department of Molecular Genetics, Kurume University, Fukuoka, JPN

**Keywords:** angiogenesis, chronic wound, extracorporeal shockwave therapy, tissue regeneration, wound healing

## Abstract

Background: Wound healing remains a major challenge in orthopedic surgeries involving thin soft-tissue injuries, such as calcaneal fractures and open wounds. Recent advancements, such as negative pressure wound therapy, have demonstrated efficacy but have practical limitations, such as patient immobility and fall risk, restricting their broader application. Extracorporeal shockwave therapy, recognized by the International Society for Medical Shockwave Treatment, has shown potential in treating complex wounds, ulcers, and burns. However, the mechanism through which extracorporeal shockwave therapy accelerates wound healing is still unclear. In this study, we aimed to investigate the effects of extracorporeal shockwave therapy on wound healing using a rat model.

Methods: We examined the effects of extracorporeal shockwave therapy on wound healing in a Sprague-Dawley rat model. Extracorporeal shockwave therapy was applied to the wound area using a cylindrical device, and histological evaluations, including immunohistochemical staining and real-time polymerase chain reaction analysis, were conducted to assess the effect of extracorporeal shockwave therapy on angiogenesis and tissue repair.

Results: Although no significant differences were found in wound contraction or vascular endothelial growth factor R2 (VEGFR2), CD31, or transforming growth factor-β1 (TGF-β1) expression, the histological analysis revealed increased vascularization and tissue regeneration in the extracorporeal shockwave therapy-treated group compared with those in the control group. On day 3, the number of blood vessels and area of CD31-positive cells were significantly higher in the extracorporeal shockwave therapy group than in the control group, with a continued increase in TGF-β1-positive cell count by day 7.

Conclusions: These findings suggest that extracorporeal shockwave therapy may enhance early wound healing by promoting angiogenesis and collagen production. Further research is needed to optimize extracorporeal shockwave therapy protocols and investigate their long-term effects on wound healing.

## Introduction

In orthopedics, wound repair is often challenging in surgeries involving thin, soft-tissue injuries, such as those observed in calcaneal fractures and open fracture treatments [1]. Wound healing is particularly difficult because bones and joints are highly susceptible to infection. In some cases, soft-tissue damage is so extensive that a skin graft may be required. However, skin flaps are not commonly employed as a primary treatment strategy in orthopedic surgery. Therefore, our goal is to manage severe wounds without using the skin flap technique, whenever possible.

In recent years, wound healing therapies, such as negative pressure wound therapy, have been advancing [2]. However, negative pressure wound therapy can limit a patient's mobility and activity, and the use of tubes can increase the risk of falls. Therefore, its use in small wounds is limited.

Extracorporeal shockwave therapy (ESWT) is currently employed for various urological [3,4], musculoskeletal [5], and cardiac [6] conditions. The ESWT guidelines [7] by the International Society for Medical Shockwave Treatment (ISMST) formally recognized difficult-to-treat skin wounds, ulcers, and burns as standard indications for ESWT. In a clinical study conducted by Wang et al., ESWT was compared with hyperbaric oxygen therapy for the treatment of diabetic foot gangrene, and ESWT was found to be effective [8]. Additionally, favorable outcomes have been reported with the use of ESWT in the treatment of ulcers caused by varicose veins [9]. Furthermore, positive results have been observed with ESWT in the treatment of wounds in diabetic rat models [10]. These findings suggest that ESWT may contribute to advancing wound therapy for post-operative soft-tissue complications.

The wound healing process comprises four phases: hemostasis, inflammation, proliferation, and tissue remodeling [11]. During the proliferative phase, growth factors such as vascular endothelial growth factor (VEGF) and transforming growth factor-β (TGF-β) are secreted. These factors are known to promote angiogenesis, tissue remodeling, tissue repair, and stimulate collagen synthesis [12]. ESWT is thought to stimulate the secretion of VEGF and TGF-β1, increase collagen-producing factor movement to the wound, and promote wound healing [13,14]. This facilitation of mechanical signaling by ESWT is referred to as mechanotransduction [15]. However, the precise mechanism through which ESWT promotes wound healing remains unclear.

The aim of this study was to investigate the effect of ESWT on acute wound healing in a rat model. We hypothesized that ESWT promotes angiogenesis and tissue repair by increasing the secretion of growth factors in the wound microenvironment.

## Materials and methods

Generation of a rat model of wound healing

**Figure 1:**
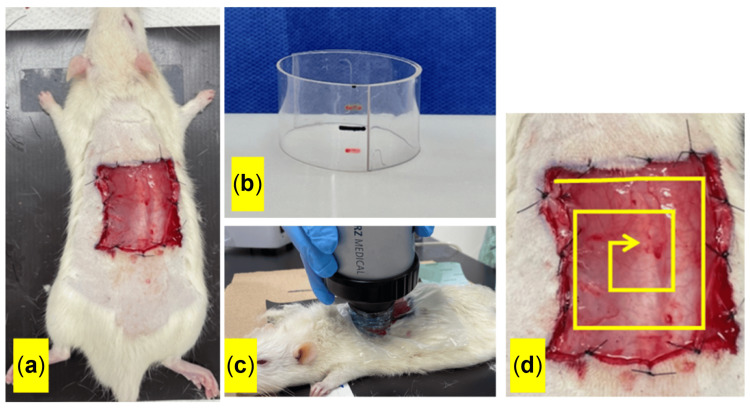
A rat wound healing model. A rectangular skin incision (a). A cylindrical device (b) was used to concentrate shockwave energy onto the wound surface (c). Applying ESWT in a spiral pattern across the wound area (d). ESWT, extracorporeal shockwave therapy. Courtesy: ImageJ software (https://imagej.net/ImageJ)

Ten-week-old male Sprague-Dawley (SD) rats (RRID: RGD_734476) were subjected to inhalation anesthesia with isoflurane. The dorsal area of the rats was meticulously shaved and demarcated with a square measuring 4 cm × 5 cm. Longitudinal skin incisions were made along the marked rectangle, and all skin layers were excised. A stay suture was made at the wound margin (Figure 1a). The study is compliant with the ARRIVE 2.0 reporting guidelines.

ESWT protocol

Isoflurane was used to anesthetize the wounded rats, and a urethane film was applied to cover the wound. Immediately after wound creation, ESWT was conducted using a Duolith SD1® focused shockwave (SW) system (Storz Medical AG, Tagerwilen, Switzerland), with a depth of focus adjusted using a 15-mm stand-off distance. A custom cylindrical device was specifically designed and constructed to concentrate the ESWT energy on the superficial layers of the wound (Figure 1b). This device was fabricated by shaping a vinyl chloride resin sheet into a 15-mm-high cylinder and was filled with ultrasound gel prior to ESWT.

In an in vitro study, Nishida et al. applied ESWT at various energy levels to vascular endothelial cells and examined VEGF mRNA expression, reporting the highest expression at 0.09 mJ/mm² [13]. Based on their findings, we delivered a total of 2,500 ESWT shots at an energy level of 0.1 mJ/mm² (Figure 1c). ESWT was performed in a spiral pattern from the periphery to the center of the wound, covering the entire wound area (Figure 1d).

Following irradiation, the wound was covered again with urethane film to prevent external interference. An Elizabethan collar was employed to prevent the rats from removing the film.

Wound area

Fourteen 10-week-old SD rats were used to establish a wound model. These rats were divided into the following two groups: SW and control (C) groups (n = 7 each group). The SW group was subjected to ESWT following the protocol described above, whereas the control group did not receive any treatment. The wound area was measured at three time points - immediately after ESWT exposure (day 0) and on days 3 and 7. To facilitate accurate measurements, the wound was covered with tracing film, and the wound edges were marked. Subsequently, wound images were captured using a digital camera under standardized conditions. The acquired images were then imported into ImageJ software 1.53c (an open‐source Java image processing program inspired by the National Institutes of Health Image, https://imagej.net/ImageJ) to determine the wound margin area. The reduction rate in the wound area was calculated by comparing the area on days 3 and 7 with that on day 0.

Molecular biological and histological evaluations

A wound model was established using 28 10-week-old (SD) rats, which were divided into SW and C groups (n = 14 in each group). The SW group was subjected to ESWT, as per the protocol described above. Three days after ESWT, tissue samples measuring 1 cm (length) × 5 mm (width) were collected from the wound margins of seven rats each from the SW and control groups. Tissue collection from the wound margins was performed to observe the wound healing process. Seven days after ESWT, tissue samples were similarly collected from the wound margins of the remaining 14 rats (seven rats each from the SW and control groups). One part of the collected tissue samples was frozen at −80°C for quantitative polymerase chain reaction (PCR) analysis, whereas the other was fixed in 10% neutral buffered formalin (#062-01661; Wako) for 24 h and subsequently subjected to paraffin embedding for immunohistochemical evaluation.

Real-time PCR analysis to measure mRNA levels

The frozen tissue sections were used to assess the expression of angiogenesis-related genes Pecam1 (CD31) and Kdr (VEGFR2), and TGF-β1, involved in collagen production and tissue repair. cDNA was synthesized from the total RNA (1 µg) extracted from the frozen tissue using Sepazol-RNA I Super G (Nacalai Tesque, Kyoto, Japan). Real-time PCR was performed on a StepOnePlus Real-Time PCR System (Thermo Fisher Scientific, Waltham, MA, USA; RRID: SCR_015805). The FastStart Universal SYBR Green Master Mix (Roche Diagnostics, Mannheim, Germany) was used for cDNA amplification. Each standard well contained pGEM-T Easy vectors (Promega, Madison, WI, USA) harboring standard cDNA fragments. Relative mRNA levels were calculated using the expression level of rat ribosomal protein S18 (Rps18), a housekeeping gene, to correct any bias introduced during RNA isolation, degradation, or differential reverse transcription. All primers used to measure TGF-β1, Pecam1 (CD31), and Kdr (VEGFR2) expression levels were purchased from Takara Bio Inc. (Siga, Japan).

Hematoxylin and eosin and immunohistochemical staining

For histological evaluation, the collected skin samples were fixed using 4% paraformaldehyde/PBS (FUJIFILM Wako Pure Chemical Co., Tokyo, Japan) and embedded in paraffin. The paraffin-embedded sections were cut into 6-µm-thick sections and subjected to hematoxylin and eosin and immunohistochemical staining. Hematoxylin and eosin staining was performed using the Hematoxylin and Eosin Stain Kit (ScyTek Laboratories, Logan, UT, USA), according to the manufacturer’s instructions. Immunohistochemical staining was performed using the following specific antibodies: anti-CD31 antibody (1:2,000) (Abcam Cat# ab182981; RRID: AB_2920881), TGF-β1 rabbit polyclonal antibody (1:400) (Proteintech Cat# 21898-1-AP; RRID: AB_2811115), and VEGF receptor 2 (D5B1) rabbit monoclonal antibody (1:800) (Cell Signaling Technology Cat# 9698; RRID: AB_11178792). Antigen retrieval (autoclaving at 105°C for 10 min) using DAKO Target Retrieval Solution (Agilent Technologies, Santa Clara, CA, USA) was performed prior to single-labeling immunohistochemistry using the high polymer method (Histofine MAX-PO (MULTI), Cat# 21300AMZ00321000; Nichirei Co., Tokyo, Japan; RRID: AB_2920810). The visualization of bound peroxidase was achieved following a 5-10 min reaction in ImmPACT DAB Peroxidase Substrate solution (Vector Laboratories, Newark, CA, USA). We confirmed that there was no staining on the control sections, wherein the primary antibody was absent. Histological analysis was performed using Olympus cellSens Software (Olympus Co.; RRID: SCR_014551) on three randomly selected fields of view from each slide. Data were evaluated using values from three randomly selected regions of interest (ROIs) per slide. The percentage of positive area was calculated based on the area of the ROI. Vessels were evaluated as the number of vessels per ROI by measuring the number of lumens surrounded by positive cells.

Statistical analysis

Results are presented as mean ± standard error of the mean for each group. Comparisons between groups were performed using the analysis of variance with the Bonferroni post-hoc test. Results with P < 0.05 were considered statistically significant.

## Results

Wound area

**Figure 2:**
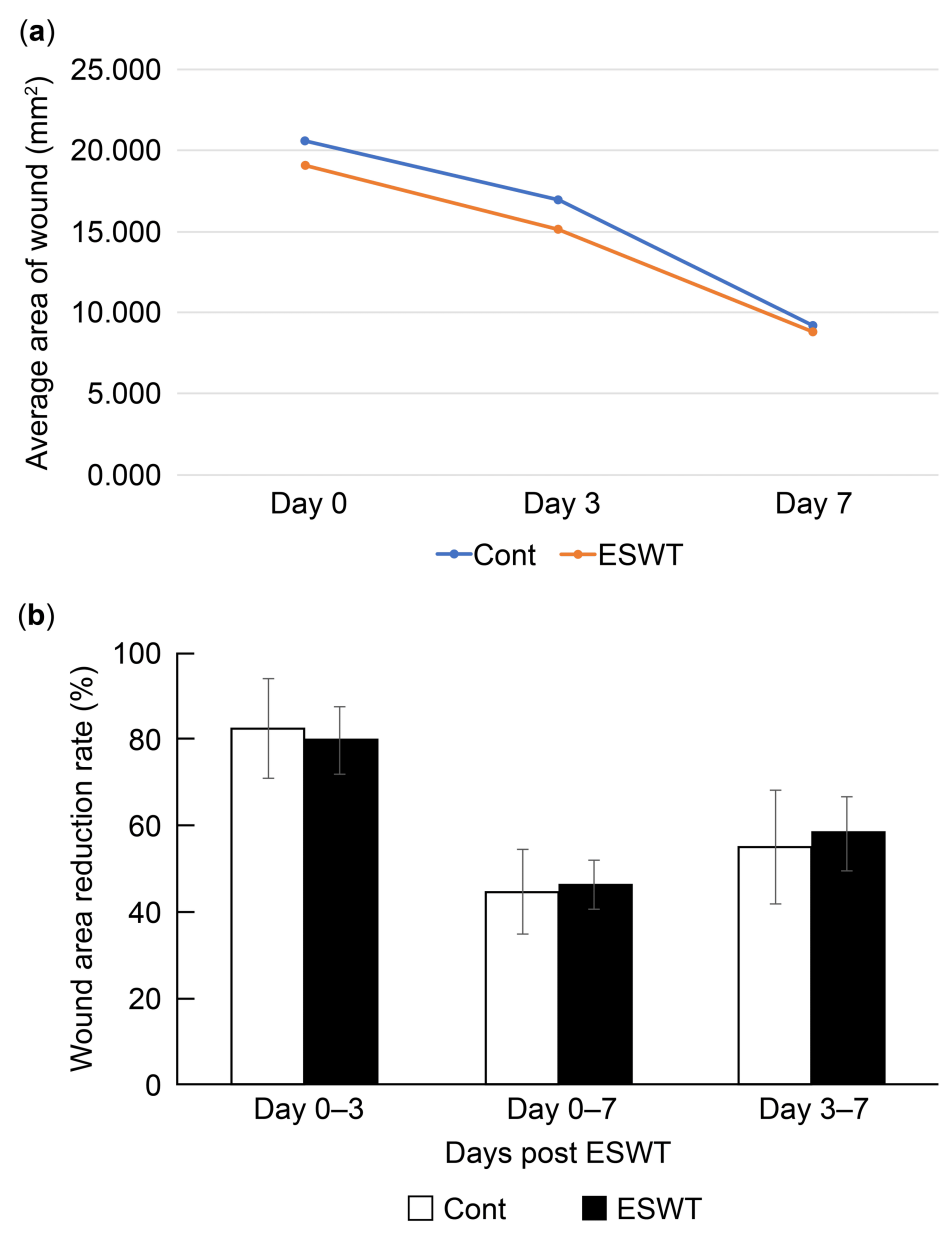
Wound area evaluation. Both control (C) and shockwave-treated (SW) groups exhibited a progressive reduction in wound area from days 0 to 7 (a). Notably, no significant difference was observed in the rate of wound area reduction between the C and SW groups during the following evaluation periods: days 0–3, 3–7, or 0–7 (b).

The wound areas decreased progressively over time in both the C and SW groups (P > 0.05, for 0-3, 3-7, and 0-7 days; Figure 2a). There was no significant difference in the rate of wound area reduction between the SW and C groups during the following intervals: days 0-3, 3-7, and 0-7 (Figure 2b).

Real-time PCR analysis

**Figure 3:**
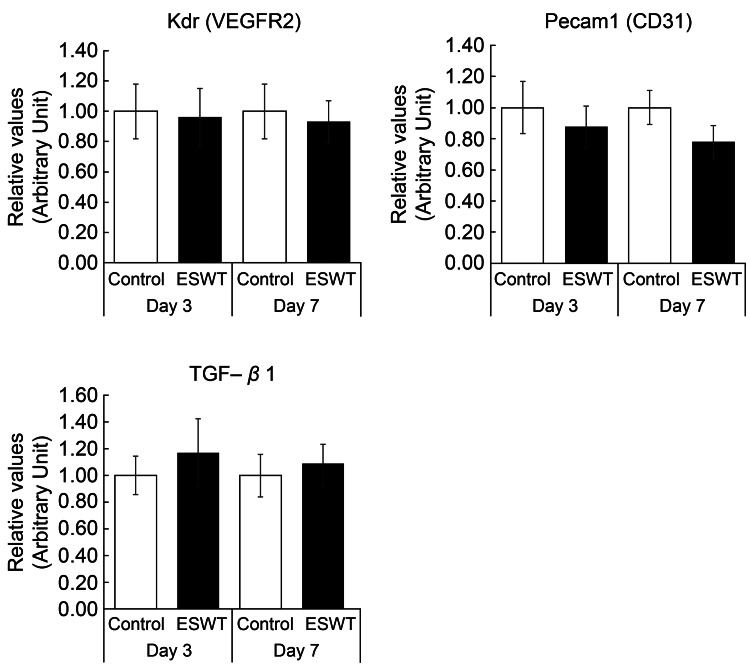
Evaluation of VEGFR, CD31, and transforming growth factor-β1 (TGF-β1) expression using real-time polymerase chain reaction (PCR). Real-time PCR analysis was performed to measure the mRNA levels of these genes. Kdr (VEGFR2) and Pecam (CD31) expression levels were evaluated to assess angiogenesis, whereas TGF-β1 expression was assessed to evaluate tissue repair. There were no significant differences in Pecam1 (CD31) and Kdr (VEGFR2) expression levels between the control (C) and shockwave-treated (SW) groups on days 3 and 7. Similarly, no significant difference was observed in TGF-β1 expression between the groups. VEGF: vascular endothelial growth factor

There was no significant difference in the expression level of the VEGFR2 (Kdr) gene between the C and SW groups on either day 3 (P = 0.88) or 7 (P ＝ 0.77); for Pecam1 (CD31), the mean value was lower in the SW group than in the C group, but the difference was not significant (day 3: P = 0.56, day 7: P ＝ 0.16). On the contrary, TGFβ1 expression was higher in the SW group on average than in the C group, although this difference was not significant (day 3: P = 0.57, day 7: P ＝ 0.70). (Figure 3).

Hematoxylin and eosin and immunohistochemical staining

**Figure 4:**
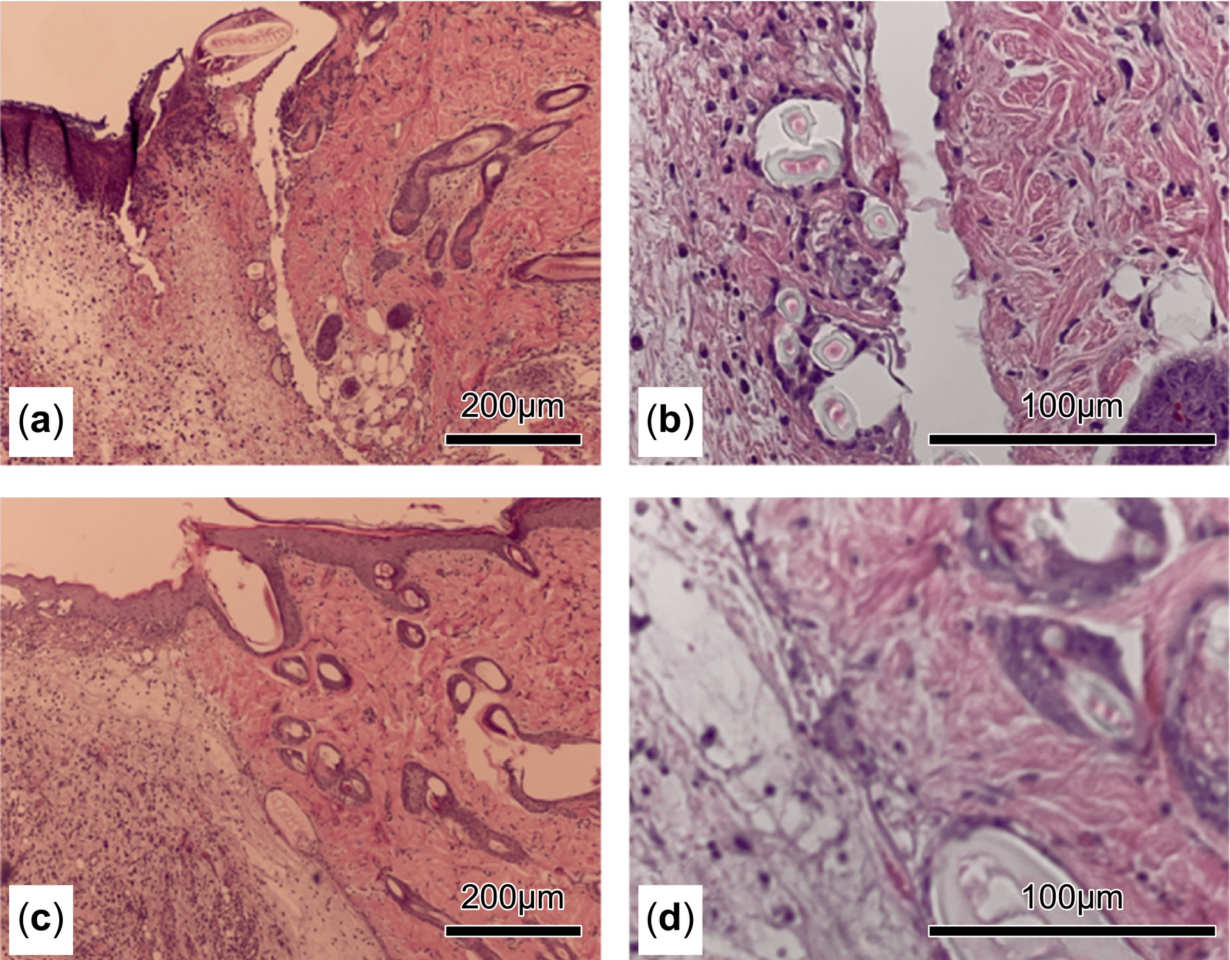
Hematoxylin and eosin staining was performed on day 3. Examination of the wound boundary showed a higher abundance of luminal structures in the shockwave-treated (SW) group (a, b) than in the control (C) group (c, d). The presence of blood cells within these luminal structures strongly suggests that they are blood vessels (b).

The hematoxylin and eosin staining results on day 3 revealed a greater abundance of luminal structures at the border of the wound in the SW group than in the C group. These luminal structures were labeled as blood vessels owing to the presence of blood cells within them (Figures 4a-4d).

**Figure 5:**
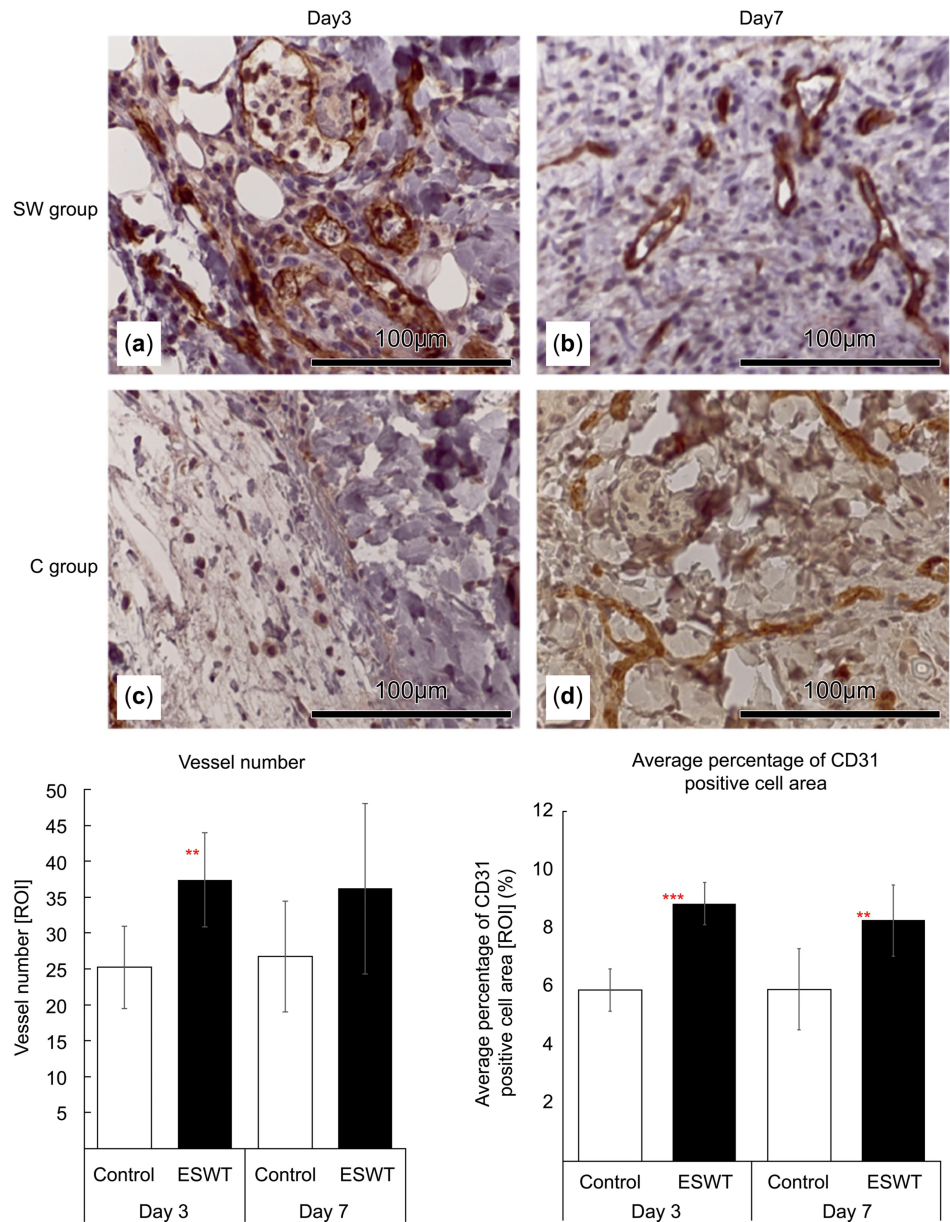
Immunohistological evaluation of CD31 expression. On day 3, the shockwave-treated (SW) group (a) exhibited a noticeable increase in CD31-positive cell area and vessel count compared to the control (C) group (b). Similarly, on day 7, both SW (c) and C (d) groups showed an increase in the number of CD31-positive cells. Quantitative analysis revealed a significant increase in CD31-positive cell area in the SW group compared with that in the C group on both days 3 and 7. Furthermore, the number of blood vessels was significantly higher in the SW group than in the control group on day 3, and a less marked difference was observed on day 7.

On day 3, the SW group exhibited substantially higher CD31-positive cell area and number of blood vessels than the C group. Similarly, on day 7, both the SW and C groups displayed increased CD31-positive cell counts compared with those on day 3 (Figures 5a-5d). The quantitative analysis demonstrated an increase in the CD31-positive cell area in the SW group compared with that in the C group on both days 3 (P < 0.01) and 7 (P < 0.01). Additionally, the number of vessels was notably higher in the SW group than in the control group on day 3 (P < 0.01); however, this difference became insignificant by day 7 (P > 0.05).

**Figure 6:**
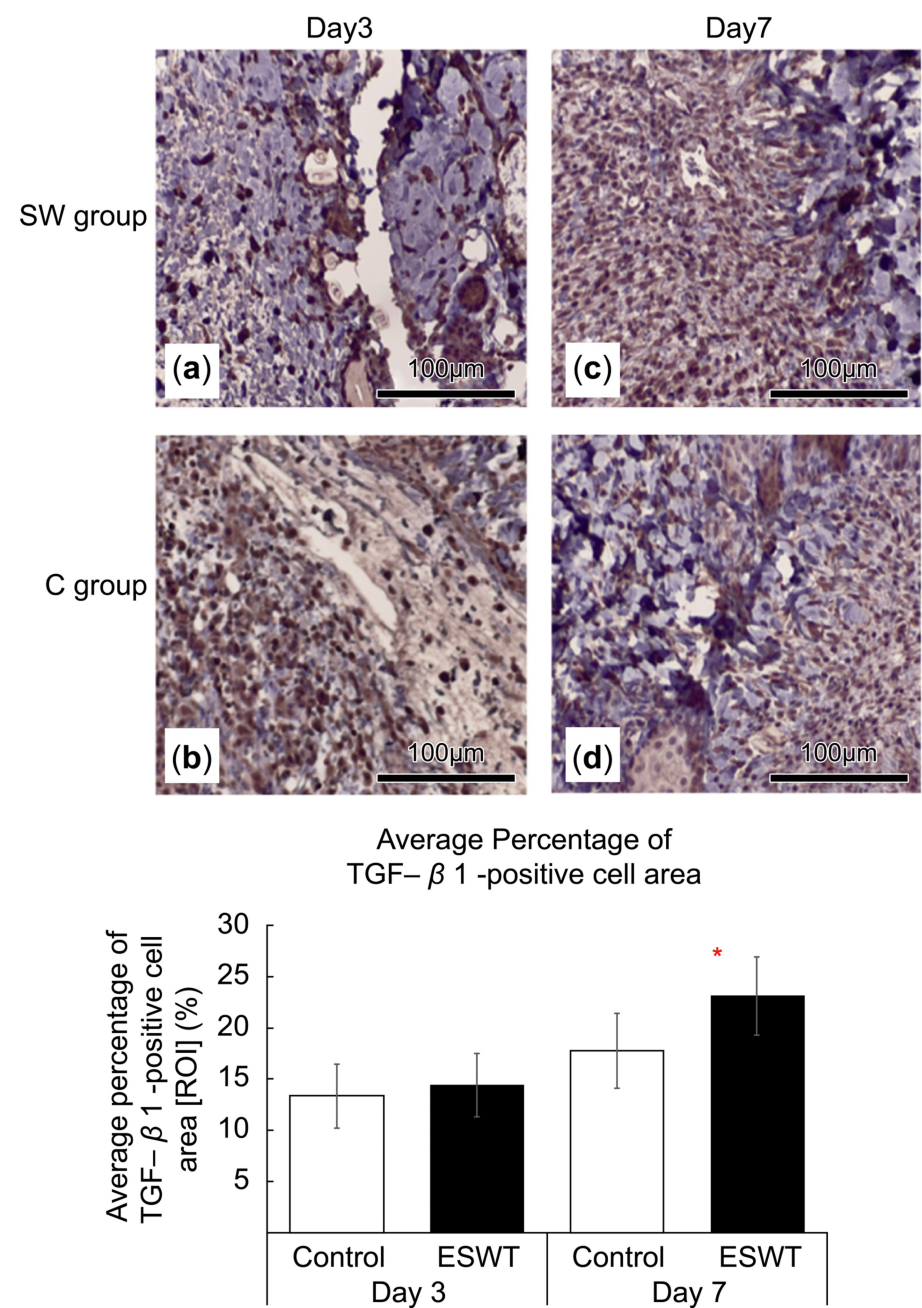
(a-d) Immunohistological evaluation of transforming growth factor-β1 (TGF-β1). Day 3 and 7 TGF-β1 staining results were obtained for the shockwave-treated (SW) and control (C) groups. Overall, the SW group exhibited a higher number of TGF-β1-positive cells than the C group on day 7. To quantitatively assess this, the TGF-β1-positive cell area was measured. On day 3, no significant difference in TGF-β1-positive cell area was found between the C and SW groups. However, on day 7, the SW group had a significantly higher area than the C group.

The SW group had a higher number of TGF-β1-positive cells than the C group on day 7 (P < 0.05; Figure 6). Hence, the TGF-β1-positive cell area was measured to quantify this observation. On day 3, no significant difference was observed between the C and SW groups (P > 0.05). However, on day 7, a significant increase was observed between the SW and C groups (Figures 6a-6d).

## Discussion

In this study, we did not observe a significant increase in the expression of VEGFR2, CD31, and TGF-β1. However, we observed a greater abundance of luminal structures at the marginal border of the wound in the SW than in the C group in hematoxylin and eosin histological staining. In addition, the number of vessels and percentage of CD31-positive cells in the SW group were significantly higher than those in the control group on day 3. The average percentage of CD31- and TGF-β1-positive cells was significantly higher in the SW group than in the control group on day 7. The expression of mRNAs induced by mechanotransduction increased within a few hours [15]. Therefore, we found no significant difference in the PCR analysis results between the SW and C groups. However, our results suggest that ESWT promotes angiogenesis and tissue repair.

Wound healing can be conceptually divided into four distinct processes or phases: hemostasis, inflammation, proliferation, and tissue remodeling [12]. During the proliferative phase, hypoxia serves as a stimulus for hypoxia-inducible factor (HIF) and COX-2 expression, both of which facilitate the release of growth factors (i.e., VEGF) and promote angiogenesis. Furthermore, fibroblasts respond to platelet- and macrophage-derived signaling molecules (i.e., TGF-β) to increase collagen production. Additionally, TGF-β plays a pivotal role in promoting myofibroblast differentiation during the remodeling phase, consequently facilitating wound contraction [12]. One of the mechanisms underlying the action of ESWT on skin wounds involves the angiogenic effect triggered by a phenomenon known as “cavitation effect” [13]. When ischemic endothelial cells are exposed to low-power ESWT, the “cavitation effect” arises from the generation and subsequent disappearance of numerous intracellular microbubbles. This effect exerts shear stress on vascular endothelial cells, thereby stimulating the secretion of angiogenesis-promoting factors such as VEGF and SDF-1, and vasodilating factors such as endothelial nitric oxide synthase (eNOS) [13]. Moreover, low-intensity ESWT induces cytokine (i.e., TGF-β1) release, which promotes collagen production, ultimately enhancing wound healing by increasing the tensile strength of the skin [13]. Therefore, the number of vessels and percentage of CD31- and TGF-β1-positive cells in the SW area were significantly higher than those in the control group in our study.

Kuo et al. created wounds in diabetic rats and irradiated them with ESWT [10]. They evaluated blood flow using a laser Doppler and reported an increase in blood flow three days after ESWT and a decrease at 10 days [10]. Similarly, in our study, peak ESWT-mediated enhancement of histological angiogenesis occurred three days after ESWT; however, no differences in gene expression levels were observed on day 3 between the SW and C groups. Zins et al. created wounds in normal and diabetic mice and subjected them to ESWT. They found that PECAM1 (CD31) expression was significantly higher on day 2 in normal mice that received a single dose of ESWT than in those that did not receive ESWT [16]. This difference between the groups decreased by day 7 [16], which is consistent with the hypothesis that changes in gene levels occur before day 3. These results suggest that ESWT induces neovascularization at the border of the wound margin.

Our study showed no significant difference in the area of TGF-β-positive cells between groups on day 3. However, by day 7, the SW group exhibited a larger area than the control. Consistent with these findings, Yang et al. reported a significantly higher area of TGF-β1-positive cells in diabetic rats treated with ESWT than in untreated controls on day 7 [14]. Similarly, Berta et al. observed enhanced TGF-β expression and collagen production in mechanically stimulated fibroblasts following ESWT [17]. In our study, the increase in CD31-positive cells on day 3 and TGF-β-positive cells on day 7 in the SW group suggests that ESWT promotes angiogenesis, recruiting platelets, macrophages, and fibroblasts, which may elevate TGF-β1 production, with a notable increase by day 7. Nonetheless, the PCR analysis did not reveal significant differences in TGF-β1 expression between the SW and control groups on days 3 or 7. This finding could be because the mRNA expression in the control group reached the level in the SW group within one day, as mechanotransduction-induced mRNA expression often occurs within hours [15]. Alternatively, the multifaceted role of TGF-β across different wound healing phases - from inflammation to remodeling - may account for these findings. Furthermore, as tissue samples were collected from the wound margin, which includes both injured and unaffected skin, the results may reflect an amalgamation of distinct wound healing stages.

Limitations

This study has certain limitations. First, different ESWT protocols (e.g., varying energy levels and irradiation frequencies) were not compared. Second, foundational evidence regarding the cylindrical device fabricated with the vinyl chloride resin was insufficient. Third, the efficacy of ESWT for infected and chronic wounds remains uncertain, as only acute wounds were evaluated in this study. Fourth, RT-PCR findings were not evaluated immediately after ESWT, and the gene expression of growth factors related to angiogenesis and tissue repair was not evaluated in this study. ESWT stimulates the secretion of angiogenic and growth factors, including VEGF, eNOS, and TGF-β1 [13,17], which may enhance its suitability for wound healing applications. ESWT comprises two main types: focused shockwave (fSW) therapy and radial shockwave (rSW) therapy. Although rSW can induce effects similar to those of fSW, such as cavitation [18], it delivers lower energy and has a limited effective range of only a few millimeters. Regarding the protocol for fSW, Nishida et al. reported that the application of fSW to vascular endothelial cells resulted in the highest VEGF gene expression at an energy flux density of 0.09 mJ/mm² [13]. In contrast, there is currently no established evidence for the optimal energy settings of rSW therapy for promoting angiogenesis or tissue repair. Therefore, we conducted our study using fSW. Moreover, as our facility did not have access to an rSW device, we were unable to evaluate its efficacy in the present study.

## Conclusions

This study demonstrated the therapeutic effect of ESWT in a rat wound model. Evaluation of wound area, real-time PCR analysis, and immunohistological staining on days 0, 3, and 7 after ESWT showed no significant differences in wound area and the expression levels of the VEGFR2 (Kdr), Pecam1 (CD31), and TGF-β1 genes between the SW and C groups. However, the CD31-positive cell area on days 3 and 7 after ESWT was significantly higher in the SW group than in the C group. The area of TGF-β1-positive cells was significantly higher in the SW group than in the C group on day 7 after ESWT. These results suggest that ESWT may promote tissue repair by stimulating early angiogenesis in wounds. Further studies are warranted to explore optimal ESWT parameters, such as the type of ESWT, irradiation frequency, energy levels, and other treatment protocols, as well as the long-term effects of ESWT.
